# Three Varieties of Membranous Anginas Produced by Microörganisms Other Than the Klebs-Löffler Bacillus and Their Sanitary Significance

**Published:** 1900-12

**Authors:** William G. Bissell

**Affiliations:** Bacteriologist, Department of Health, Buffalo, N. Y.


### THREE VARIETIES OF MEMBRANOUS ANGINAS PRODUCED BY MICROÖRGANISMS OTHER THAN THE KLEBS-LÖFFLER BACILLUS AND THEIR SANITARY SIGNIFICANCE.

By WILLIAM G. BISSELL, M. D.,
Bacteriologist, Department of Health, Buffalo, N. Y.

THAT the diphtheria bacillus is not the only microörganism capable of producing a pseudo-membranous inflammation, is a well established fact. Prior to the inauguration of the more exact microscopic methods for the determination of a diphtheritic infection, there were several varieties of pseudo-membanous inflammation of the tonsils classes as "diphtheria" which, by recent research, have been conclusively demonstrated to be produced by microörganisms other than the Klebs-Löffler bacillus. The appearance of the membranes in these conditions is not dissimilar to that produced by the Klebs-Löffler bacillus, and the constitutional disturbance may be such as not to be capable of differentiation from that usually attending a severe case of diphtheria.

The microörganisms in question are the streptococcus pyogenes, micrococcus of sputum septicemia, which belongs to the bacteria, and oidium albicans, a member of the fungi group. A Klebs-Löffler infection has been shown to be capable of wide variances in its clinical manifestations, and these organisms can, likewise, produce illness having a clinical picture varying from the most mild sore throat to a fatal disease.

In the experience of the Bureau of Bacteriology in this city, the oidium albicans has never produced a membranous condition of the tonsils that has resulted fatally; but instances of streptococcus and sputum septicemic infections have frequently occurred, followed in several instances by death of the patient. These conditions cannot be classed with the contagious diseases in the true sense of the term, for though they are capable of transmission by direct inoculation, they do not appear to be communicable.

In the early part of March, 1896, the first investigation was carried on relative to a membranous condition of the tonsil, producing a severe clinical disturbance which resulted fatally. Dr. J. H. Potter, of Buffalo, submitted a culture, and examination revealed the presence of short-chained streptococci and staphylococci. Considering the illness to be diphtheria, he submitted a second culture, examination of which resulted as in the former instance. A small piece of membrane was found on the swab accompanying the second culture outfit, and examination revealed the presence of streptococci almost to the exclusion of any other organism. A diagnosis of a streptococcus tonsilar infection was made. On the seventh day of the disease, the patient, a child, died, the result of a general toxemia. Diphtheritic antitoxin had been administered on two different occasions without the slightest beneficial effect.

This being the first reported instance in Buffalo of death resulting from a streptococcus tonsilar infection, the facts in the case were submitted to Dr. Herman Biggs, director of the New York bacteriological health laboratories, requesting information as to whether similar instances had occurred in New York. Dr. Biggs replied that they had had several deaths from streptococcus toxemias, the tonsils being the seat of the primary invasion.

The following statement of Dr. Thomas Bagley, Buffalo, N. Y., contains an interesting account of a severe streptococcus tonsilar infection:

February 11, 1900, I was called to attend a boy 10 years of age. His parents stated that he had not been feeling well for about a day, complaining particularly of a sore throat. On examination, I found both tonsils enlarged, the right having the ordinary appearance of a follicular tonsilitis, the left presenting in its central portion a very suspicious membranous patch. The temperature at this time, was 104° F., pulse, 160, patient somewhat delirious and extremely nervous. On the second day, the general condition was worse, the temperature being 105° F; pulse, 170.

The right tonsil still retained the appearance of a follicular tonsilitis, but the left tonsil was completely covered with a dark grayish membrane resembling that usually seen in a severe case of tonsilar diphtheria. Becoming alarmed at the condition, 2,000 units of diphtheritic antitoxin serum were injected, and a culture sent to the Department of Health for confirmation of diagnosis. On the following day a report was received that the culture did not reveal anything typical of diphtheria, but requested that a second culture be submitted. In the meantime, the membranous patch had extended to the pharynx, into the posterior nasal passages, entirely covered the soft palate, and seemed inclined to extend forward. The right tonsil was completely covered with a membrane

The case, clinically, resembled diphtheria. A second culture was submitted, and, at this time, I was successful in obtaining a portion of the membrane which was, likewise, delivered to the bacteriological laboratory. The patient's condition was rapidly becoming more serious. The following morning, a report was received that there were no diphtheria bacilli in the culture. Examination of the membrane revealed streptococci to be present almost to the exclusion of other organisms.

On the fifth day of the disease, the temperature ranged from $103\frac{1}{2}°$ to $105°$ F; pulse, 120 to 170. There was no change in the appearance of the throat. Local treatment had included the use of strong solutions of the subsulphate of iron, a mixture of carbolic acid and glycerine, several applications of Löffler's solution, and continual use of peroxide of hydrogen. As the patient's condition was such that death seemed inevitable, and owing to the fact that streptococci predominated in the membrane, on the eighth day of the disease, as a last resort, 10 c.c. of antistreptococcus serum was employed. To my surprise, the boy's condition began to improve. The membrane commenced to exfoliate, and three days following, the uvula and left tonsil were entirely cleared of membrane. In a few days, the throat assumed a healthy appearance. The patient has made a good recovery.

An instance occurred, April 16, 1897, in the experience of the Health Department's laboratory, where the micrococcus of sputum septicemia was concerned in the production of a tonsilitis. Dr. Carlton R. Jewett submitted a culture from the throat of Miss B. The patient was extremely ill with a condition, considered to be tonsilar diphtheria. Examination of the culture did not reveal the presence of the Klebs-Löffler bacillus. Reluctant to believe the condition to be other than diphtheria, Dr. Jewett requested that the bacteriologist in person visit the case.

On the following day, Dr. Bissell, under the direction of Dr. Jewett, made several cultural inoculations and also succeeded in obtaining a small portion of the membranous exudate, which, at this time, covered the greater portion of both tonsils and portions of the uvula and naso-pharynx. Examination of the cultures resulted as in the former instance. Examination of the membrane revealed the micrococcus of sputum septicemia in abundance.

The patient died about the tenth day of the disease. Drs. C. G. Stockton, H. R. Hopkins and Roswell Park had been called in consultation and were unanimous in the statement that as regards the macroscopic appearances of the tonsils and the clinical symptoms, the illness could not be distinguished from a severe case of diphtheria. Diphtheritic antitoxin of a reliable make had been administered at the onset of the disease without the slightest improvement. There is no question but that this patient died the result of a toxemia, due to the micrococcus of sputum septicemia, the primary lesions being confined to the tonsils, uvula and naso-pharynx.

As regards the oidium albicans, many instances have occurred in the experience of the laboratory where the physician has recorded the diagnosis on the culture examination blank as diphtheria and the culture revealed an abundance of the "thrush" fungus. The membrane, in these conditions, has been noted as being present on the tonsils, the sides of the cheeks, the uvula, and, in one instance, a cast of the upper portion of the esophagus was submitted for inspection.

The following conclusions can be drawn from the above reported cases:

1. The streptococcus pyogenes and the micrococcus of sputum septicemia can produce membranous anginas, accompanied by physical disturbances sufficient to result in death.

2. The oidium albicans produces pseudo-membranous exudates easily mistaken for a Klebs-Löffler inflammation.

3. The only positive means of determining a Klebs-Löffler infection is by microscopic methods.

4. From the sanitary standpoint, as regards quarantine, anginas due to the streptococcus pyogenes, micrococcus of sputum septicemia and the oidium albicans, require little consideration.

143 Elmwood Avenue.

